# Responses of *Calligonum leucocladum* to Prolonged Drought Stress Through Antioxidant System Activation, Soluble Sugar Accumulation, and Maintaining Photosynthetic Homeostasis

**DOI:** 10.3390/ijms26094403

**Published:** 2025-05-06

**Authors:** Fang Yang, Guanghui Lv

**Affiliations:** 1 School of Ecology and Environment, Xinjiang University, Urumqi 830017, China; yf13579863579@163.com; 2 Key Laboratory of Oasis Ecology, Ministry of Education, Urumqi 830017, China; 3 Xinjiang Jinghe Observation and Research Station of Temperate Desert Ecosystem, Ministry of Education, Jinghe 833300, China

**Keywords:** drought stress, growth adaptability, physiological response, protein expression

## Abstract

Desert shrubs play an important role in the stability of arid and fragile desert ecosystems. However, despite their significant ecological importance, limited research has been performed on shrub drought tolerance strategies at the morphological, physiological, and molecular levels. Therefore, this study focused on the typical desert shrub, *Calligonum leucocladum*, and analyzed its morphology, physiology, and protein expression under two different habitats: moist low-salt and arid low-salt. The results indicate that drought stress inhibited the growth of *C. leucocladum*, leading to significant reductions in its plant height, base diameter, and crown width by 14.93%, 49.57%, and 48.49%, respectively. Drought stress triggered a 30% decline in stomatal conductance, whereas homeostasis was observed in net photosynthesis, intercellular CO₂, and transpiration. The soluble sugar content significantly increased by 13.43%, while the starch, soluble protein, and proline content significantly decreased by 20.32%, 10.67%, and 55.61%, respectively. In addition, under drought stress, membrane peroxidation products, reactive oxygen species metabolites, and antioxidant enzyme activities significantly increased. Weighted gene co-expression network analysis revealed 40 proteins that were significantly enriched in the photosynthesis and oxidative phosphorylation pathways through KEGG enrichment analysis. In addition, *C. leucocladum* maintains photosynthetic homeostasis by enhancing PSII repair (PsbE, PsbL, PsbH) and electron transfer chain efficiency (PetD, nad 2, nad 9), thereby compensating for the insufficient carbon dioxide supply caused by stomatal limitation. This study integrated multidimensional data from morphology, physiology, and proteomics to reveal that *C. leucocladum* drives a coupled network of photosynthesis, antioxidant, and carbon metabolism through chloroplast translation reprogramming. It maintains photosynthetic homeostasis and osmotic balance under a 30% decrease in stomatal conductance, elucidating the cross-scale regulatory strategy of desert shrubs adapting to extreme drought.

## 1. Introduction

Drought stress, as a crucial environmental stress factor in desert ecosystems, has impacted over one-third of terrestrial ecosystems globally, significantly restricting the accumulation and development of plant biomass [1,2]. The heterogeneity of seasonal precipitation in desert areas leads to drastic fluctuations in soil moisture [3], further intensifying the survival pressure on plants and prompting desert vegetation to evolve unique resource allocation strategies. Analyzing plants’ long-term drought tolerance mechanisms can provide a theoretical basis for species selection and adaptive afforestation in arid areas for ecological restoration.

In desert habitats, plants primarily respond to drought stress by altering their morphological structure and physiological responses, thereby adjusting their ecological adaptation [4]. This adaptation encompasses alterations in plant height, root morphology, leaf succulence, photosynthesis, osmotic regulation, reactive oxygen species (ROS) metabolism, and antioxidant defense systems [5,6]. Research has shown that water deficits inhibit cell expansion, resulting in a reduced leaf area, decreased internode elongation, and lower plant height [7]. Drought primarily impacts photosynthesis and associated physiological metabolic processes. Numerous studies indicate that drought reduces the photosynthetic capacity of leaves, subsequently disrupting photosystem II (PSII) within the photosynthetic apparatus [8]. On the other hand, damage to PSII can affect carbon fixation, reduce energy production, and lead to excessive ROS production [9]. To mitigate the damage inflicted by ROS on the membrane system, the accumulation of proline and other osmoregulatory substances increases, while the enzyme activity involved in the ROS clearance system correspondingly rises, thereby maintaining the balance between ROS production and clearance in plants [10]. In addition, plants can maintain a balance between drought tolerance and growth by altering their root structure and stomatal closure [11]. However, the stress response mechanisms underlying these physiological and biochemical changes have not yet been systematically elucidated in *C. leucocladum*.

The core mechanism by which plants respond to environmental stress lies in the dynamic regulation of proteins [12]. Research has shown that key enzymes in the antioxidant system, such as ascorbic acid/glutathione metabolism-related proteins, exhibit a 3–5-fold increase in expression in desert *Phragmites communis*, effectively mitigating drought-induced oxidative damage [13]. The NAC domain protein and LEA protein of *Populus trichocarpa* are specifically induced by drought and are involved in cell dehydration protection [14]. Under drought stress, the expression of proteins related to oxidative phosphorylation and PSII is upregulated in the desert plant *Xanthoceras sorbifolium* Bunge, accompanied by an increase in the abundance of PSI subunit proteins. This reveals the selective characteristics of photosystem damage and the ecological specificity of oxidative phosphorylation system recombination [15]. This clearly demonstrates the significant importance of proteomics in deciphering the molecular mechanisms regulating various biological processes in plants. Therefore, by studying the role of these induced proteins in response to drought stress, we can gain insights into pathways related to drought tolerance. In addition, physiological indicators and protein functions exhibit dynamic interactions during plant stress responses [2]. The former are driven by changes in protein expression/activity, while the synthesis and degradation of the latter are also inversely regulated by physiological states.

Ebinur Lake in Xinjiang, China, is a typical desert ecosystem characterized by low precipitation, water scarcity, and plants constantly threatened by drought stress [16]. *Calligonum leucocladum* is a deciduous shrub of the Polygonaceae family, belonging to the genus *Calligonum*. It is a typical arid desert plant found in active sand dunes and stable sand fields in the arid desert of Ebinur Lake and can tolerate extreme drought and cold [17]. Because of its fast growth, dense branches, and easy reproduction, it has become widely used as a windbreak, to fix sand, and in ecological restoration projects in western China, and it plays a key role in stabilizing desert ecosystems [18]. As key species in ecological engineering in arid regions, the drought resistance of *Calligonum* species directly determines their sand fixation ability and community stability. However, there is currently a notable gap in our understanding of the synergistic relationship between their drought resistance mechanisms and ecological functions. Existing research has primarily focused on the physiological characterization of drought resistance in plants of the *Calligonum* genus, yet it has not clarified how their protein expression regulatory networks drive their drought resistance.

Therefore, this study focused on *C. leucocladum* growing in natural environments. We selected two habitats with different soil moisture: a moist low-salt habitat and an arid low-salt habitat. Firstly, we compared and analyzed the morphological and physiological characteristics of *C. leucocladum* in two different habitats and explored its morphological and physiological adaptation strategies for long-term drought tolerance. Then, quantitative proteomics techniques were employed to analyze the protein expression changes in *C. leucocladum* in two different habitats, aiming to identify the key metabolic pathways and regulatory proteins that respond to drought. Finally, by integrating morphological and physiological data, the long-term drought tolerance adaptability of *C. leucocladum* was systematically elucidated. These data will aid in understanding the protein regulatory patterns in xerophytes and their adaptation strategies to long-term drought stress in desert ecosystems.

## 2. Results

### 2.1. Effect of Prolonged Drought on C. leucocladum Growth

**Figure 1:**
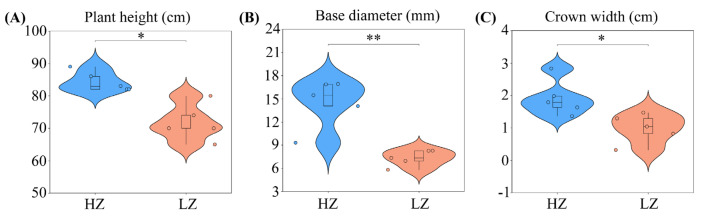
Morphology of *C. leucocladum* in low-salt environments: HZ (humid) and LZ (arid): (**A**) Plant height; (**B**) base diameter; (**C**) crown width. * *p* < 0.05, ** *p* < 0.01. Error bars represent standard deviation of the mean (*n* = 5).

Plants adapt to varying water environments through morphological adjustments [16]. Long-term drought significantly inhibits the growth of *C. leucocladum* (Figure 1). Specifically, the plant height, base diameter, and crown width decreased by 14.93%, 49.57%, and 48.49%, respectively.

### 2.2. Effect of Prolonged Drought on C. leucocladum Physiology

**Figure 2:**
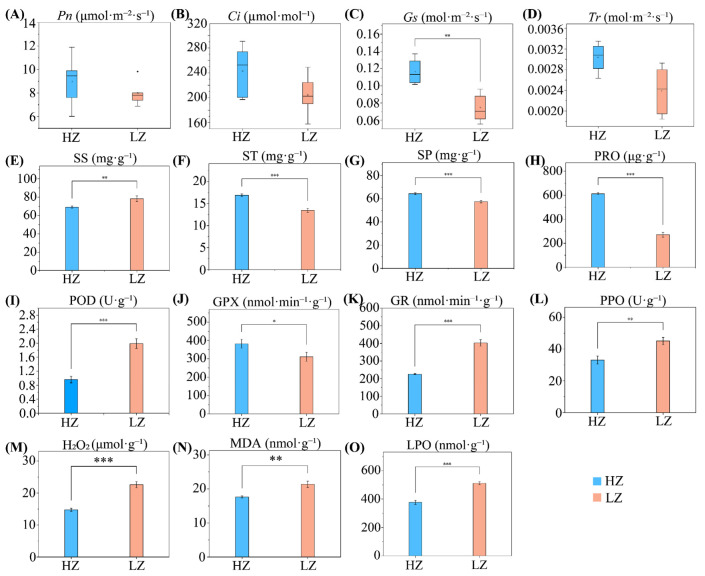
Physiological characteristics of *C. leucocladum*: (**A**) Net photosynthetic rate (*Pn*); (**B**) intercellular carbon dioxide concentration (*Ci*); (**C**) pore conductance (*Gs*); (**D**) transpiration rate (*Tr*); (**E**) soluble sugar (SS); (**F**) starch (ST); (**G**) soluble protein (SP); (**H**) proline (PRO); (**I**) peroxidase (POD); (**J**) glutathione peroxidase (GPX); (**K**) glutathione reductase (GR); (**L**) polyphenol oxidase (PPO); (**M**) hydrogen peroxide (H_2_O_2_); (**N**) malondialdehyde (MDA); and (**O**) lipid peroxidation (LPO). HZ, humid, low-salt environment; LZ, arid, low-salt environment. * *p* < 0.05, ** *p* < 0.01, and *** *p* < 0.001. Error bars represent the standard deviation of the mean (*n* = 3).

To gain a deeper understanding of the impact of drought on the physiological characteristics of *C. leucocladum*, we analyzed changes in photosynthesis, osmotic adjustment substances, and antioxidant enzyme activity. The findings revealed that drought stress significantly reduced *Gs* (*p* < 0.01) (Figure 2A–D). This suggests that photosynthetic inhibition is primarily due to stomatal limitation. Plants’ drought tolerance mechanisms involve osmotic regulation and ROS metabolism, which mitigate the damage caused by adverse conditions [19]. Drought stress significantly activated the antioxidant enzyme system (with increased activities of POD, GR, and PPO, *p* < 0.01), yet it failed to completely prevent membrane lipid peroxidation (the MDA, LPO, and H_2_O_2_ content significantly increased, *p* < 0.01). This indicates that the rate of ROS generation exceeded the clearance capacity, resulting in cell membrane damage. Additionally, drought stress notably reduced the levels of ST, SP, and PRO (*p* < 0.001), whereas the SS content significantly increased (Figure 2E–H). This suggests that drought hinders protein synthesis or accelerates its degradation. *C. leucocladum* may prioritize allocating nitrogen resources towards SS synthesis rather than PRO accumulation, balancing carbon and nitrogen metabolism requirements.

### 2.3. The Trade-Off Between Growth and Physiology in C. leucocladum

**Figure 3:**
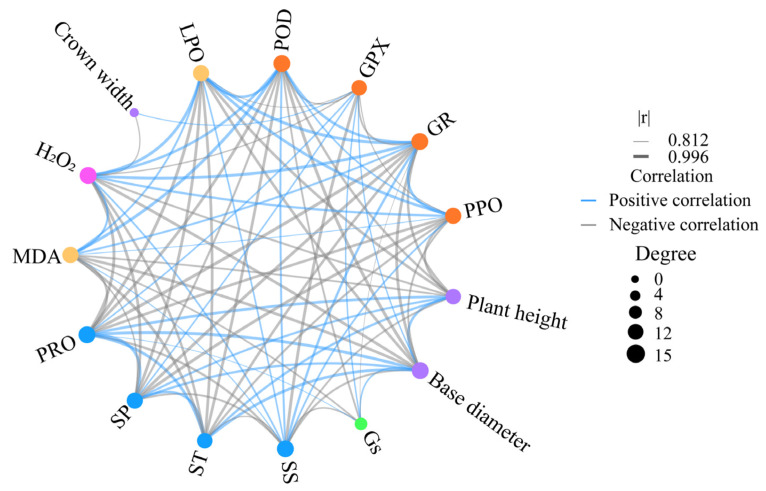
Correlation network between growth and drought tolerance traits in *C. leucocladum*. The larger a point, the greater the degree of connection (there are more indicators related to it). The thicker a line, the greater the correlation.

To reveal the morphological and physiological interactions of *C. leucocladum* under drought stress, 15 indicators significantly affected by drought stress were selected for correlation analysis. The results reveal a significant negative correlation between morphological and physiological characteristics in most cases (Figure 3, Table S1). Plant height and basal diameter were significantly and positively correlated with ST, SP, and PRO (r > 0.93, *p* < 0.01) and significantly and negatively correlated with SS, POD, GR, PPO, H_2_O_2_, MDA, and LPO (r < −0.90, *p* < 0.05). SP, ST, and PRO were strongly and positively correlated (r > 0.98, *p* < 0.001) and significantly and negatively correlated with POD, GR, PPO, H_2_O_2_, MDA, and LPO (r < −0.88, *p* < 0.05). SS was significantly and positively correlated with GR, POD, PPO, H_2_O_2_, MDA, and LPO (r > 0.84, *p* < 0.05), and negatively correlated with ST, SP, PRO, GPX, and *Gs* (r < −0.86, *p* < 0.05). Additionally, *Gs* was significantly and negatively correlated with POD, GR, and MDA (r < −0.81, *p* < 0.05) and positively correlated with GPX and PRO (r > 0.82, *p* < 0.05).

### 2.4. Protein Expression in C. leucocladum in a Drought Environment

**Figure 4:**
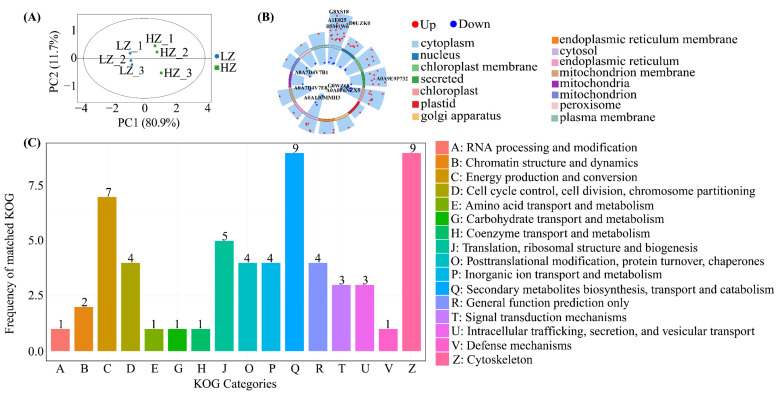
Proteomics analysis: (**A**) PCA analysis; (**B**) DEP volcano map; (**C**) classification of KOG functional annotations for DEPs. Low-salt environment: HZ, humid; LZ, drought.

TMT proteomics revealed 233 proteins after screening for the number of unique peptide segments belonging to a protein (retaining ≥ 1 unique peptide segment) (Table S2). Principal component analysis (PCA) was performed on all samples to understand the overall differences and changes between and within groups. The two principal components (PC1 and PC2) explained 80.9% and 11.7% of the variation, respectively, indicating a significant difference in protein content between plants from the HZ and LZ habitats (Figure 4A). Following screening criteria, 85 DEPs (62 upregulated and 23 downregulated) were identified (Table S3), distributed mainly in “cytosis”, then “chloroplasts” (Figure 4B). Additionally, DEPs were mainly associated with secondary metabolite biosynthesis, a class of proteins related to transport and catabolism, the cytoskeleton, and energy production and conversion, all of which were upregulated (Figure 4C, Table S4).

### 2.5. Screening of Key Drought-Tolerant Proteins Using WGCNA

**Figure 5:**
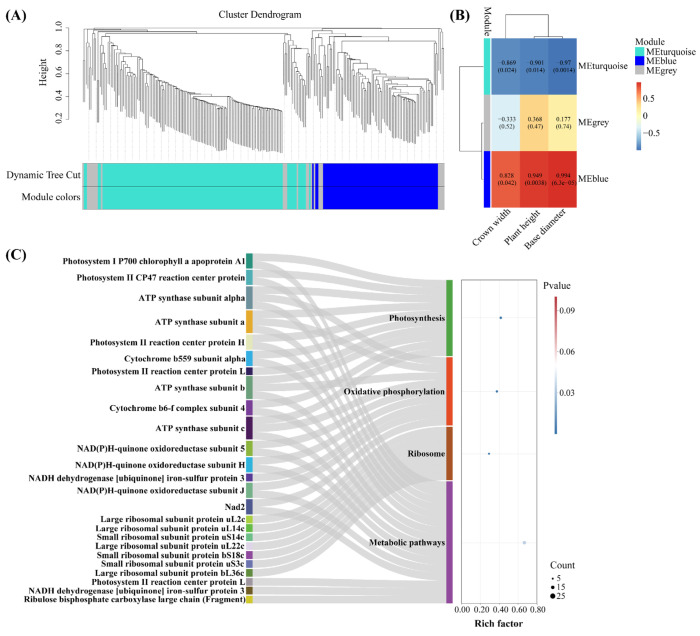
WGCNA module for proteins in *C. leucocladum* under drought stress: (**A**) Hierarchical clustering tree based on topological overlap and dissimilarity; (**B**) module–trait relationships; (**C**) KEGG analysis of DEPs.

To build a co-expression network, an expression matrix was constructed based on 233 identified proteins (Section 2.4) for WGCNA analysis. We constructed a hierarchical clustering tree based on the correlation coefficients between protein expression levels. Subsequently, proteins were categorized according to their expression patterns based on weighted correlation coefficients. The proteins were divided into three expression modules, among which the MEturquoise and MEblue modules were closely correlated with the growth index (Figure 5A,B). Under drought stress, proteins specifically expressed in *C. leucocladum* were mainly concentrated in MEturquoise (Figure S1A). To identify key drought-tolerant regulatory proteins, highly correlated proteins were screened from the MEturquoise and MEBlue modules to construct a related network (Figure S1B); a total of 40 key drought-tolerant proteins were identified (Table S5). KEGG enrichment analysis revealed that the “photosynthesis pathway” (map00195) was most significantly affected by drought stress, followed by “oxidative phosphorylation” (map00190), “ribosome” (map03010), and “metabolic pathways” (map01100) (Figure 5C).

### 2.6. DEPs Involved in Photosynthesis and Oxidative Phosphorylation Under Drought Stress

**Figure 6:**
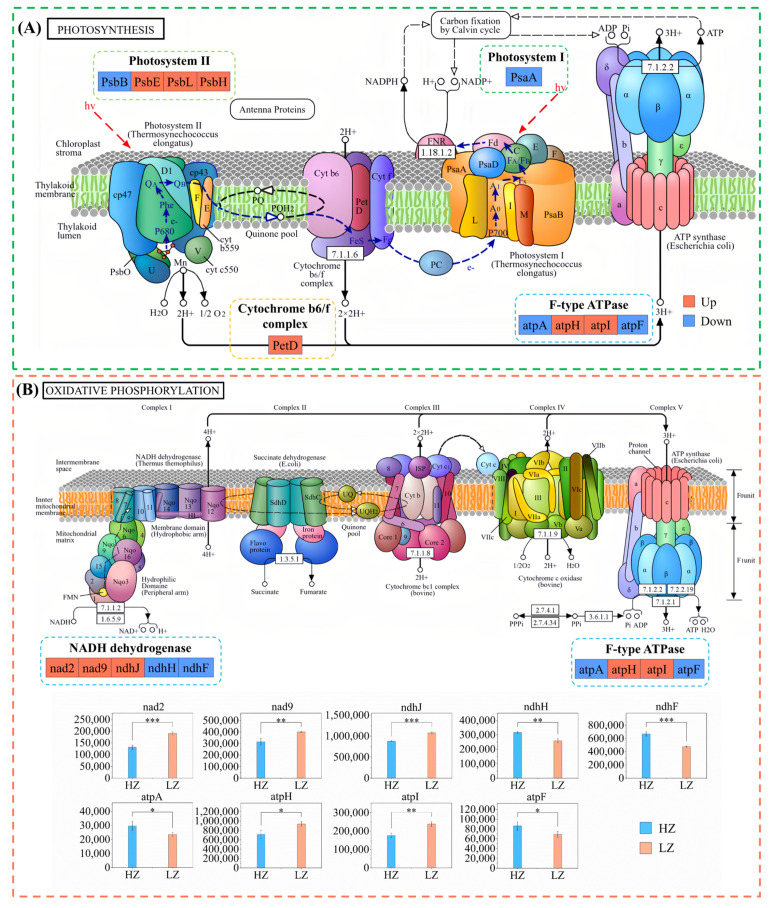
Photosynthesis and oxidative phosphorylation regulation spectra. Regulation pathway of (**A**) photosynthesis and (**B**) oxidative phosphorylation. Box color: blue, protein downregulation; red, protein upregulation. HV represents light energy. * *p* < 0.05, ** *p* < 0.01, and *** *p* < 0.001. Error bars represent standard deviations of the mean (*n* = 3).

KEGG pathway enrichment analysis revealed “photosynthesis” (map00195) to be the most significantly enriched pathway (*p* = 2.33 × 10^−12^), followed by “oxidative phosphorylation” (map00190) (*p* = 1.96 × 10^−8^. A protein expression regulation mechanism diagram was constructed based on the enriched DEPs in this pathway (Figure 6). Among these, photosystem II proteins (PsbE, PsbL, and PsbH), cytochrome b6/f complex protein (PetD), and F-type ATPase protein (atpH and atpI) were upregulated in LZ plants, whereas photosystem I protein (PsaA) was downregulated (Figure 6A, Table S6). Under drought stress, most proteins involved in oxidative phosphorylation (nad 2, nad 9, ndhJ, atpH, and atpI) were upregulated (Figure 6B). These results contradict the physiological results, indicating that a compensatory mechanism may occur in *C. leucocladum* under drought stress.

## 3. Discussion

### 3.1. Physiological Mechanisms Behind Response to Drought Environments in C. leucocladum

In arid habitats, maintaining cellular water balance through osmotic regulation is a crucial physiological adaptation strategy for plants [20]. In this study, SS exhibited significant accumulation in *C. leucocladum*, aligning with the osmotic regulation patterns observed in most xerophytes [16,21]. Research has shown that the accumulation of SS enhances plant drought resistance through a dual mechanism. On one hand, it promotes water absorption by reducing cell osmotic potential [22], and on the other hand, it participates in ROS clearance [23]. However, it is worth noting that, unlike in typical xerophytes [24], the PRO and SP contents of *C. leucocladum* significantly decreased under drought stress. This phenomenon may be associated with species-specific adaptation strategies.

Previous studies have demonstrated that a significant accumulation of PRO typically occurs during severe protein metabolism disorders [25]. Meanwhile, studies have shown that drought stress significantly reduces SP content by inhibiting ribosome biosynthesis pathways, which aligns closely with the dynamic changes observed in SP during this study [26]. However, in this study, the expression of proteins related to ribosome biosynthesis was mostly upregulated. On one hand, drought stress may activate the stress pathway (indicated by the upregulation of calmodulin expression), prioritizing the synthesis of functional proteins (such as the upregulation of superoxide dismutase and glutathione S-transferase expression). On the other hand, the accumulation of ROS and changes in osmotic pressure caused by drought may disrupt the folding environment of newly formed proteins, leading to the formation of insoluble aggregates of unfolded or misfolded proteins instead of existing in soluble form. Therefore, the metabolic characteristics of *C. leucocladum* may reflect its evolved energy-saving strategy, which prioritizes the strengthening of the SS metabolic pathway over initiating the highly energy-consuming PRO/SP synthesis system.

During drought, the amount of ROS in plants increases, leading to the activation of antioxidant enzyme protective mechanisms to maintain metabolic balance [27]. The significant accumulation of H_2_O_2_ (ROS metabolite) and POD, GR, and PPO enzyme activities confirmed this. Under stress conditions, the accumulation of ROS in plants can lead to membrane peroxidation, which negatively affects plant growth [2]. MDA is a product of LPO and reflects the degree of membrane lipid peroxidation [28]. We report that MDA and LPO contents significantly increased in LZ habitat plants, indicating that *C. leucocladum* experienced membrane damage under prolonged drought conditions. Despite the activation of antioxidant enzymes to eliminate excessive ROS, drought conditions still damage plant cells [29].

### 3.2. Trade-Offs Between Growth and Physiology in Response to Drought in C. leucocladum

The trade-off between growth and stress resistance can be explained by energy and resource constraints; plants under stress transfer energy and resources from growth to a stress response [30]. Long-term drought stress significantly reduced the plant height, basal diameter, and crown width of *C. leucocladum* (Figure 1). This phenotypic characteristic aligns closely with the typical resource optimization strategies employed by desert plants [31]. The correlation analysis between morphology and physiology reveals that *C. leucocladum* optimizes water use efficiency through a dual strategy involving the synergistic regulation of morphology (reducing plant height and base diameter) and physiology (ST, SP, and PRO). This “growth–stress” trade-off mechanism is common in desert plants such as *Encelia farinosa* [6] and *Nitaria sibirica* [16]. Its essence lies in the redistribution of photosynthetic products from structural growth to stress defense.

The correlation analysis in this study strongly indicates that SS may play a dual role in both ROS production (SS shows a significant negative correlation with H_2_O_2_) and clearance (SS demonstrates a significant positive correlation with POD, GR, and PPO). This finding aligns with previous research indicating that SS can promote ROS production and indirectly enhance ROS clearance by stabilizing antioxidant enzyme structures [32]. Furthermore, the upregulated expression of superoxide dismutase and glutathione-S-transferase further corroborates this notion. In addition, SS negatively regulates stomatal opening during photosynthesis in *C. leucocladum*. These results suggest that SS not only serves as an osmotic regulator to maintain cell osmotic potential but also coordinates redox balance through its dual “metabolic–signaling” properties. This regulatory mode complements the sugar signaling cascade theory proposed by predecessors and provides evidence for the adaptive evolution of desert plants in the ROS homeostasis model [5,33].

### 3.3. Drought Stress Promotes Expression of Photosynthetic and Oxidative Phosphorylation-Related Proteins in C. leucocladum

*Gs* values play a crucial role in gas exchange between plants and the atmosphere [34]. Moderate stomatal closure is an adaptation to environmental stress [35]. A decrease in *Gs* reduces *Ci*, which is considered the main limiting factor for photosynthesis under a water deficit [36]. In this study, the *Gs* of *C. leucocladum* was significantly reduced, yet there were no notable changes in *Pn*, *Ci*, or *Tr*. This phenomenon challenges the traditional “stomatal limitation dominance hypothesis”. This may be attributed to the synergistic upregulation of PSII core proteins (PsbE, PsbL, and PsbH) and cytochrome b6/f complex subunits (PetD), which facilitates the formation of proton gradients in thylakoid membranes and enhances the diffusion efficiency of CO₂ in mesophyll cells [37,38]. The decoupling phenomenon between stomatal limitation and carbon assimilation serves as a crucial mechanism for *C. leucocladum* in achieving a balance between “efficient water use and sustained carbon assimilation” under drought stress.

The upregulation of PsbE, PsbL, PsbH, PetD, atpH, and atpI proteins indicated that the photosynthetic apparatus of *C. leucocladum* was stable under drought stress, supporting the hypothesis that these proteins play key roles in plant drought stress responses and tolerance. The decrease in *Pn* runs contrary to the increase in photosynthetic protein expression in *C. leucocladum* under drought stress, possibly because of a positive feedback response [38]. While oxidative stress leads to the inactivation of proteins involved in photosynthesis, the upregulation of ribosome-related proteins (rpl 2, rpl 14, rps 14, rpl 22, rps 18, and rps 3) induces the expression of photosynthesis-related proteins in *C. leucocladum* [37]. This strategy enables *C. leucocladum* to maintain photosynthesis and enhance drought tolerance by compensating for decreased *Pn*.

Oxidative phosphorylation involves the transfer of electrons from NADH or FADH_2_ to molecular oxygen to form water, coupled with ADP phosphorylation to generate ATP [39]. Drought stress typically leads to increased energy demand and respiration [40]. We report that most proteins involved in oxidative phosphorylation were upregulated, including NADH dehydrogenases, NADH ubiquinone oxidoreductases, and enzymes involved in ATP synthesis. This indicates that *C. leucocladum* may maintain its primary physiological activity and inhibit stress damage by increasing energy production. NADH ubiquinone oxidoreductases generate superoxide and H_2_O_2_, which serve as significant sources of ROS production [41]. In this study, we observed that drought stress induces upregulation of ndhJ, which subsequently enhances ROS production in *C. leucocladum*. However, mitochondria can prevent ROS production through the alternate oxidase pathway, bypassing complexes III and IV (Figure 6B) and transferring electrons directly to O_2_, thereby generating heat energy instead of ATP [42]. The downregulation of ATP biosynthesis proteins atpA and atpF indicates that the generated energy exists as thermal energy to avoid excessive ROS accumulation. Concurrently, the upregulation of atpH, atpI, nad 2, nad 9, and nadJ can maintain high-energy metabolism in *C. leucocladum* to achieve normal growth.

This study focused on the correlation among morphology, physiology, and protein expression, without exploring the dynamic changes in key enzymes involved in photosynthesis and proteins related to oxidative phosphorylation through transcriptomics and metabolomics. In the future, multiomics approaches should be utilized to further analyze the specific regulatory mechanisms of proteins related to photosynthesis and oxidative phosphorylation and systematically elucidate how *C. leucocladum* regulates the balance between photosynthesis and energy production under drought stress.

## 4. Materials and Methods

### 4.1. Research Area

The Ebinur Lake Wetland National Nature Reserve (44°30′–45°09′ N, 82°36′–83°50′ E) is situated in northwest Jinghe County, Bortala Mongol Autonomous Prefecture, Xinjiang Uygur Autonomous Region. This watershed is located deep within the Eurasian Continent. The plain region, which is far from the ocean and encircled by mountains on three sides, experiences a typical temperate continental arid climate owing to their combined influence. The annual evaporation exceeds 1600 mm, whereas the annual rainfall is approximately 100 mm [43]. Long-term drought stress has seriously affected the local ecosystem, with plants in this region primarily relying on groundwater resources for growth [16]. The primary vegetation types within the study area are small trees, shrubs, and semi-shrubs. The main plant species include *Populus euphratica*, *Haloxylon ammodendron*, *Tamarix ramosissima*, and *C. leucocladum* [44].

### 4.2. Site Layout and Plant Selection

**Figure 7:**
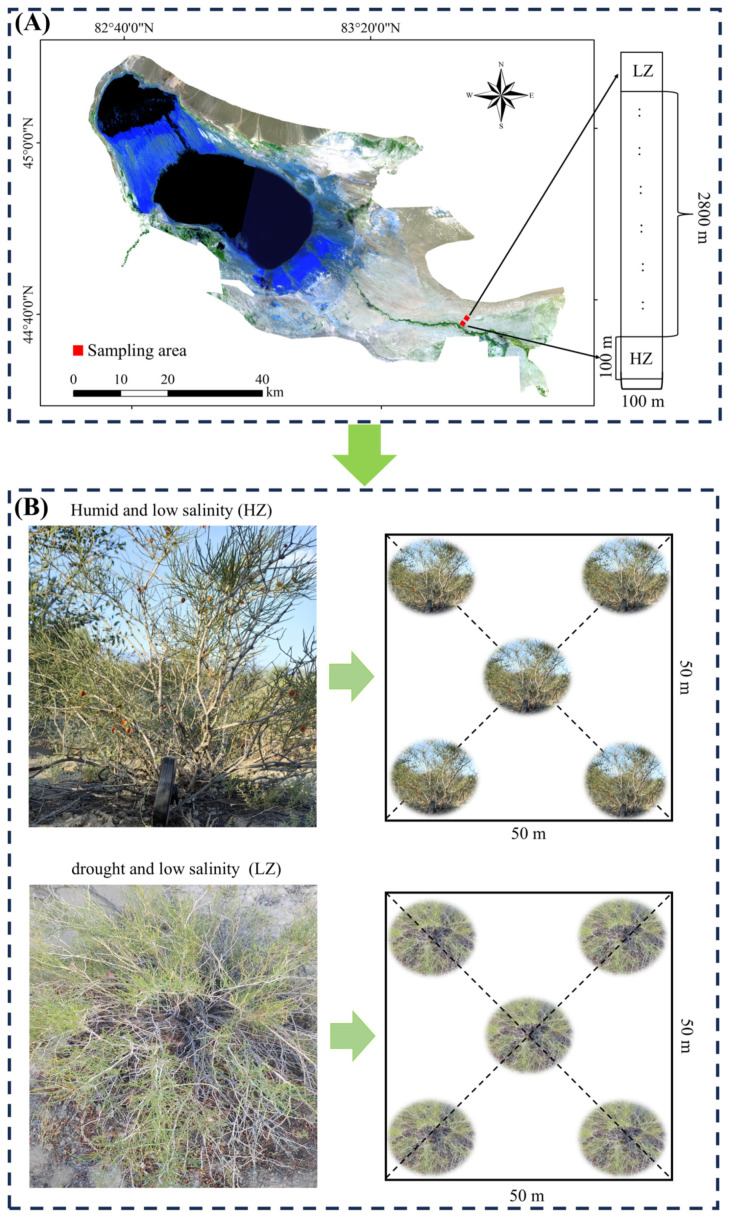
Study area and sample map: (**A**) Low-salt habitats: humid (HZ) and arid (LZ). (**B**) Plant selection.

At the Jinghe Temperate Desert Ecosystem Field Scientific Observation Station of the Ministry of Education (Xinjiang University), two 100 m × 100 m plots were established in areas with *C. leucocladum* distribution, perpendicular to the Aqikesu River. These plots were designated as a moist low-salinity habitat (HZ) and an arid low-salinity habitat (LZ), respectively, with the starting point set at the Dongdaqiao Management Station of the Ebinur Lake Wetland Nature Reserve (Figure 7A). The two sampling sites were located 2800 m apart from each other. Within each plot, a 50 m × 50 m quadrat was established, within which five healthy, similarly sized plants were sampled (Figure 7B). There is a significant water gradient perpendicular to the Aqiksu River in the desert hinterland [21,43]. The soil moisture content differed significantly (*p* < 0.05) between the HZ (7.25%) and LZ (2.11%) plots (Table S7).

### 4.3. Determining Plant Morphology

Plant height and crown width were measured using a tape measure (±0.1 cm), and plant basal diameter was measured using a Vernier caliper.

### 4.4. Determining Photosynthetic Parameters

An LI-6800 (Licor, Lincoln, NE, USA) photosynthesis measurement system was used to measure the instantaneous photosynthetic rate of leaves (gas exchange indicators) on sunny mornings between 09:00 and 11:00 h. The light quantum flux density (PPFDi) inside the leaf chamber was set to 1200, with a reference CO_2_ concentration of 400 μmol mol^−1^, a flow rate of 500 μmol s^−1^, and a leaf chamber temperature control of 30 °C. Because the leaves of *C. leucocladum* are scaly, fleshy, and approximately cylindrical, leaf diameter was measured using Vernier calipers (± 0.05 mm), and the total leaf area in the leaf chamber was calculated using the formula to calculate the surface area of a cylinder (because the leaf chamber is under single-sided illumination, the actual photosynthetic area should be half of the calculated area). The measured parameters included the net photosynthetic rate (*Pn*, μmol·m^2^·s^−1^), transpiration rate (*Tr*, mol·m^2^·s^−1^), intercellular carbon dioxide concentration (*Ci*, µmol·mol^−1^), and stomatal conductance (*Gs*, mol·m^2^·s^−1^).

### 4.5. Physiological Parameters

Leaves from the upper and middle assimilating branches of five plants in each soil environment were collected and mixed evenly, and three replicate samples were collected. For each replicate, the soluble sugar (SS) content was determined using the sulfuric acid anthrone method [45]; the starch (ST) content was determined using the perchloric acid method [46]; the soluble protein content (SP) was determined using a protein quantification kit [47], while the proline (Pro) content was measured using the ninhydrin method [48]; malondialdehyde (MDA) and lipid peroxide (LPO) were measured according to the instructions provided with the kit [49], and H_2_O_2_ was assayed using the enzyme-linked immunosorbent assay method [50]. Peroxidase (POD) catalyzes H_2_O_2_ to oxidize specific substrates and has characteristic light absorption at 470 nm [51]; glutathione peroxidase (GPX) catalyzes the oxidation of organic peroxides to reduced glutathione (GSH), producing oxidized glutathione (GSSG). Glutathione reductase (GR) catalyzes the reduction of GSSG by NADPH, regenerating GSH, while NADPH is oxidized to generate NADP^+^. GPX and GR activities were calculated by measuring the rate of light absorption reduction at 340 nm light absorption [52]. Polyphenol oxidase (PPO) activity was determined using the catechol method [53].

### 4.6. TMT (Tandem Mass Tag) Quantitative Proteomics Analysis

The samples were ground individually in liquid nitrogen and lysed with SDT lysis buffer (containing 100 mM NaCl) and a 1/100 volume of Dithiothreitol (DTT), followed by 5 min of ultrasonication on ice. After reacting at 95 °C for 8–15 min and being placed in an ice bath for 2 min, the lysate was centrifuged at 12,000× *g* for 15 min at 4 °C. The supernatant was taken and added with sufficient IAM to react for 1 h at room temperature in the dark. Then, the samples were completely mixed with 4 × the volume of precooled acetone via vortexing and incubated at −20 °C for at least 2 h. The samples were then centrifuged at 12,000× *g* for 15 min at 4 °C, and the precipitate was collected. After washing with 1 mL of cold acetone, the pellet was completely dissolved in Dissolution Buffer (DB buffer) [54]. A bovine serum albumin (BSA) standard protein solution was prepared according to the Bradford protein quantification kit instructions. Protein samples were collected, and DB protein solution was added to release protease; samples were labelled following Thermo TMT’s labeling instructions [55]. The raw data (.raw) for mass spectrometry detection were generated using a Q ExactiveTM HF-X mass spectrometer equipped with a Nanospray Flex™ (ESI) ion source. The resulting spectrum was derived from the raw files obtained through mass spectrometry. Subsequently, each run was individually searched using the library search software Proteome Discoverer 2.5 (PD, Thermo Fisher Scientific, Wuhan, China, HFX, and 480).

To improve the quality of analysis, we used PD software 2.5 to filter the results; peptide spectrum matches (PSMs) with a credibility > 99% were deemed reliable, and proteins encompassing at least one unique peptide segment were regarded as trustworthy. Only trustworthy peptides and proteins were retained, and false discovery rate (FDR) validation was performed to remove peptides and proteins with an FDR > 1% [56]. *t*-tests were performed on protein quantification results; proteins exhibiting significant quantitative differences between arid low-salt and humid low-salt conditions (*p* < 0.05, with a fold change either ≤0.83 or ≥1.2) were designated as differentially expressed proteins (DEPs) [57].

### 4.7. Statistical Analysis

The morphological and physiological indicators of *C. leucocladum* in the two different habitats were analyzed using a *t*-test, with the significance level set at α = 0.05. Pearson correlation analysis was conducted between morphological and physiological indicators using the “psych” package in R4.4.2 software, and the results were visualized using Cytoscape software 3.10.0 [58].

To identify significant differences in protein expression between arid low-salt and humid low-salt conditions, the data were subjected to logarithmic transformation and centering using R (version 4.4.2). Clusters of Orthologous Groups of Protein (KOG) and Kyoto Encyclopedia of Genes and Genomes (KEGG) databases were utilized for protein function and enrichment pathway analysis [59]. We utilized the “WGCNA” package in R4.4.2 software to conduct weighted gene co-expression network analysis [60].

## 5. Conclusions

This study systematically unveiled the multidimensional adaptation strategies of *C. leucocladum* under long-term drought stress. The research findings suggest that long-term soil moisture deficit hinders the growth of *C. leucocladum*, but it initiates the redistribution of resources towards the synthesis of osmoregulatory substances. SS, serving as a pivotal osmotic regulator, maintains cell turgor homeostasis by directing carbon metabolism flow. In addition, *C. leucocladum* effectively removes ROS by activating the antioxidant system, thereby minimizing membrane peroxidation and enhancing drought tolerance. The photosynthetic system maintains functional stability through a “stomatal limitation compensation mechanism” and ensures light energy conversion by enhancing the PSII repair efficiency and electron transfer rate. The synergistic upregulation of key proteins involved in energy metabolism bolsters ATP production driven by transmembrane proton gradients, thereby laying an energy foundation for drought response. This study is the first to elucidate that desert shrubs achieve drought adaptation through a trinity molecular network comprising “carbon allocation optimization, light protection enhancement, and energy metabolism remodeling”. This provides a theoretical foundation and a protein marker library for dissecting the post-translational modification regulatory mechanisms underlying plant drought resistance and for desert ecological restoration.

## Data Availability

The original data in this study are publicly available at ProteomeXchange (PXD058928) from https://www.iprox.cn/page/PSV023.html;?url=1734929811320QqCB (accessed on 31 October 2024) (password: vCtj).

## Supplementary Materials

The following supporting information can be downloaded at: https://www.mdpi.com/article/10.3390/ijms26094403/s1.
