# Supplementary material for: Responses of Calligonum leucocladum to Prolonged Drought Stress Through Antioxidant System Activation, Soluble Sugar Accumulation, and Maintaining Photosynthetic Homeostasis
Source: Int J Mol Sci. 2025 May 6;26(9):4403. doi: 10.3390/ijms26094403 (PMC12072819; doi:10.3390/ijms26094403)
Supplement: Supplementary file 1 [file ijms-26-04403-s001.zip › ijms-3554712-supplementary/Supplementary data/Figure S1.pdf]

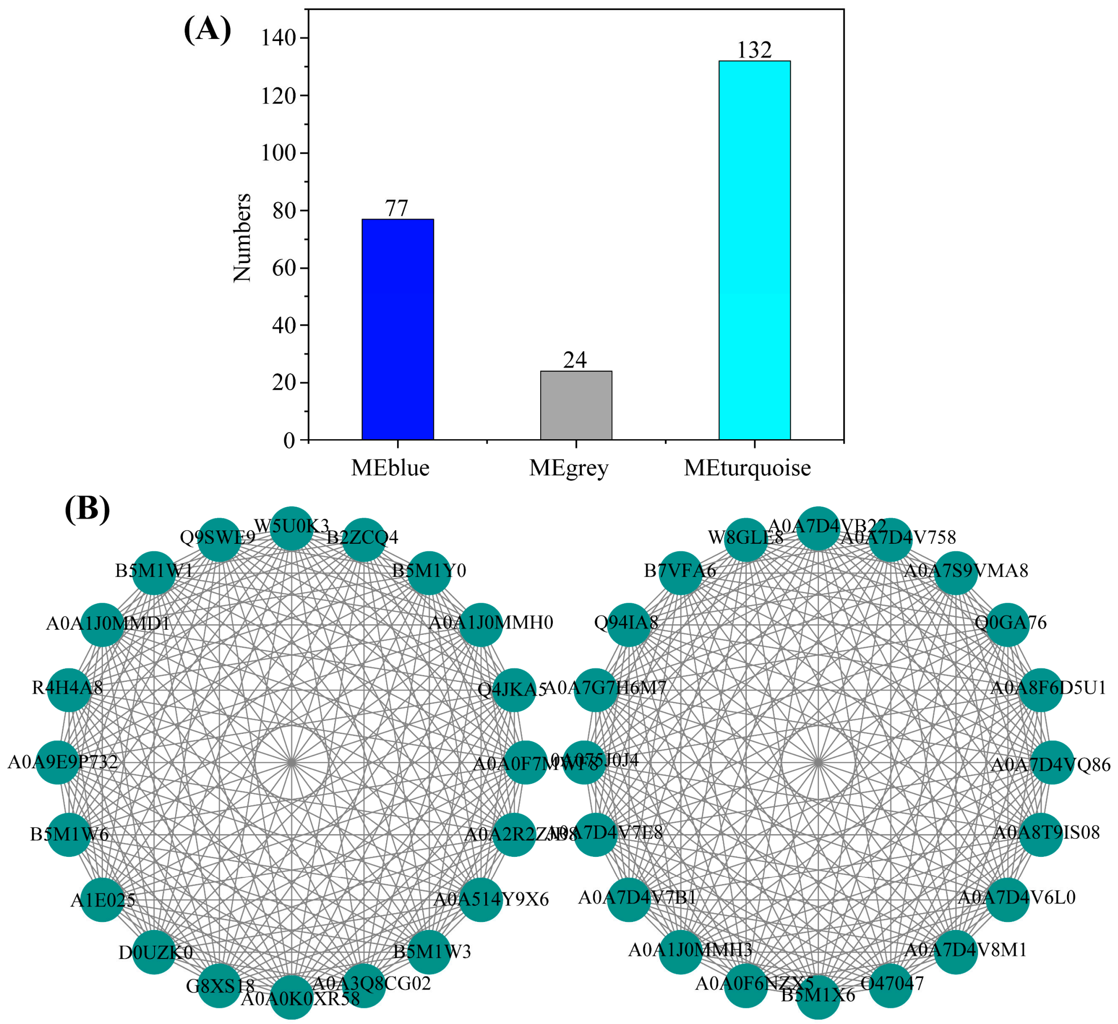

Figure S1. Construction of WGCNA module of proteins of *C. leucocladum* under drought stress. (**A**) The number of proteins in each module; (**B**) Key proteins regulatory network for drought tolerance.
