# Supplementary material for: Responses of Calligonum leucocladum to Prolonged Drought Stress Through Antioxidant System Activation, Soluble Sugar Accumulation, and Maintaining Photosynthetic Homeostasis
Source: Int J Mol Sci. 2025 May 6;26(9):4403. doi: 10.3390/ijms26094403 (PMC12072819; doi:10.3390/ijms26094403)
Supplement: Supplementary file 1 [file ijms-26-04403-s001.zip › ijms-3554712-supplementary/Supplementary data/Table S7.pdf]

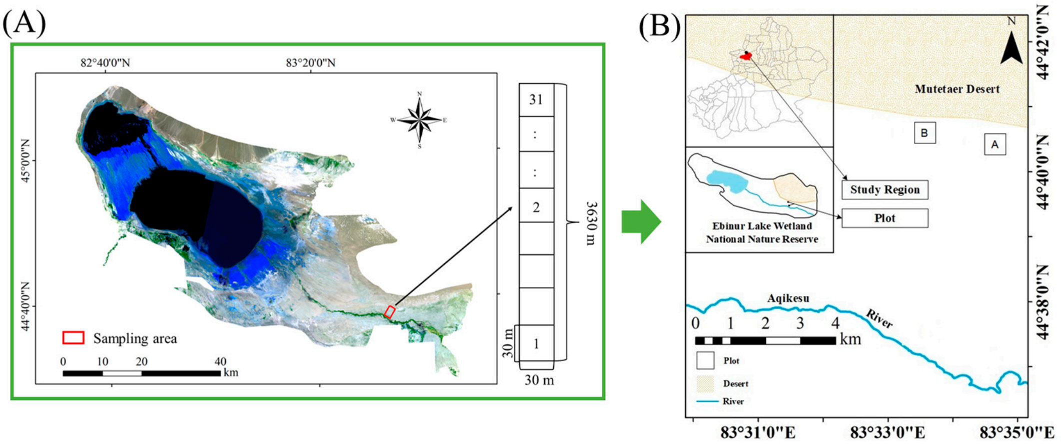

Figure S2. Detailed geographic information map of the research area. (**A**) Research sample strip; (**B**) sampling area.

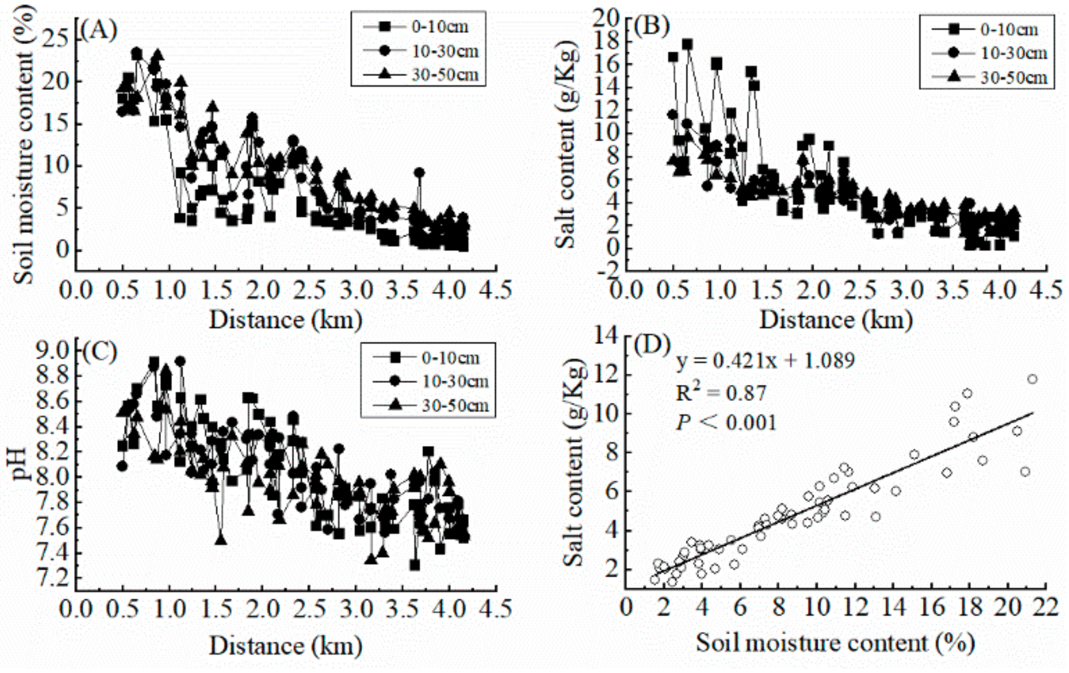

Figure S3. Characteristics of soil moisture, salinity, and pH variations from the riverbank to the desert hinterland, covering a total distance of 3630m and 31 sampling plots. (**A**) Characteristics of soil moisture content changes; (**B**) characteristics of soil salt content changes; (**C**) characteristics of soil pH changes; (**D**) the correlation between soil moisture and soil salinity. Distance refers to the distance from the riverbank. Solid squares, circles, and triangles respectively represent different depths of soil layers.

Table S1. Detailed information on soil properties in two different habitats.

Plots	SWC (%)	SSC (%)	pH
HZ	7.25a	1.18a	7.91b
LZ	2.11b	0.41b	8.09a

Note: Data represent the mean ± standard deviation (*n*=5). HZ and LZ represent moist low salt and arid low salt habitats, respectively. SWC, Soil moisture content; SSC, Soil salinity; Different lowercase letters (a, b) indicate significant differences between two habitats at the 0.05 level.
